# Missing the mark(ers): circulating endothelial cells and endothelial-derived extracellular vesicles are elevated in sickle cell disease plasma

**DOI:** 10.3389/fimmu.2024.1493904

**Published:** 2024-12-24

**Authors:** Joan D. Beckman, Ping Zhang, Julia Nguyen, Robert P. Hebbel, Gregory M. Vercellotti, John D. Belcher

**Affiliations:** Department of Medicine, Division of Hematology, Oncology and Transplantation, University of Minnesota, Minneapolis, MN, United States

**Keywords:** extracellular vesicles (EVs), endothelium, sickle cell anemia, circulating endothelial cells (CEC), soluble adhesion molecules, sickle cell pain crisis

## Abstract

Sickle cell disease (SCD) is a devastating hemolytic disease, marked by recurring bouts of painful vaso-occlusion, leading to tissue damage from ischemia/reperfusion pathophysiology. Central to this process are oxidative stress, endothelial cell activation, inflammation, and vascular dysfunction. The endothelium exhibits a pro-inflammatory, pro-coagulant, and enhanced permeability phenotype. We used flow cytometry to enumerate circulating endothelial cells (CECs, CD31+/CD45-/CD146+) in SCD and normal healthy control blood samples. Furthermore, we assessed CEC subtypes, including circulating endothelial progenitor cells (EPCs, CD31+/CD45-/CD146+/CD133+) and mature CECs (mCECs, CD31+/CD45-/CD146+/CD133-) with mCECs further subdivided into resting CECs (rCECs, VCAM-1-) and activated CECs (aCECs, VCAM-1+). As compared to healthy controls, total CECs and mCECs were elevated in SCD blood as compared to healthy control blood. Using the same markers along with size-based gating, we also used flow cytometry to enumerate endothelial-derived extracellular vesicles (EEVs) in plasma. We assessed EEV subtypes based on VCAM-1 expression, including activated EEVs (aEEVs, CD31+/CD45-/CD146+/CD133-/VCAM-1+) and resting EEVs (rEEVs, VCAM-1 negative), presumably derived from activated and resting endothelial cells, respectively. aEEVs were elevated in SCD patient plasma as compared to healthy controls. Importantly, in SCD patients, total EEVs and aEEVs were increased during self-reported pain crisis as compared to steady state. Plasma markers of endothelial cell activation including soluble E-selectin, P-selectin, VCAM-1, and ICAM-1 were elevated in SCD plasma. These data highlight strategies to detect SCD-related endothelial cell activation and demonstrate that endothelial cell activation markers may be useful to evaluate curative and non-curative therapies in SCD patients.

## Introduction

Sickle cell disease (SCD) manifests due to a single base substitution at the sixth position of the hemoglobin β chain resulting in hemoglobin S (HbS). In the deoxygenated state, HbS can polymerize, which leads to red blood cell (RBC) hemolysis, anemia, endothelial cell activation, vaso-occlusive pain crises (VOC), organ dysfunction, and early mortality (1). In 1980 Hebbel et al. demonstrated that sickle RBCs are more adherent to vascular endothelium suggesting that abnormal interactions between red cells and endothelium could be the initiating factor in the development of microvascular occlusions in SCD (2). Subsequently the vital role of the endothelial cell in SCD pathophysiology has been delineated. In SCD, endothelial dysfunction is driven by the ischemia/reperfusion (I/R) physiology that potentiates inflammation, oxidative stress, hypercoagulability, and VOCs (3).

Secondary to acute and chronic hemolysis along with I/R physiology, the SCD vasculature exhibits a pro-inflammatory, pro-coagulant, and enhanced permeability phenotype (1). Overall, vascular endothelial cells respond to over 30 different agonists (4), with markers of activation including, but not limited to, increased secretion of von Willebrand factor (VWF) and P-selectin from Weibel-Palade bodies (4), increased pro-coagulant tissue factor expression (5), and expression of adhesion molecules, such as vascular cell activation marker 1 (VCAM-1) and intercellular adhesion molecule 1 (ICAM-1) (4). Correspondingly, SCD plasma exhibits increased expression of endothelial-derived cytokines, acute-phase proteins, hypercoagulable markers, such as soluble tissue factor, and adhesion molecules, including ICAM-1, VCAM-1, E-selectin, and P-selectin (6).

Beyond soluble markers, plasma from SCD individuals has increased circulating endothelial cells (CECs), reflecting the activation state of the vessel wall (7–14). CECs include mature circulating endothelial cells (mCECs) that have detached from the blood vessel wall into the bloodstream, as part of vessel remodeling or vascular damage (15). These CECs express platelet endothelial cell adhesion molecule (PECAM, CD31+) and melanoma cell adhesion molecule (MCAM, CD146+), but do not express the leukocyte common antigen (CD45-) or the progenitor cell marker prominin-1 (CD133-). mCECs include both “activated” CECs (aCECs, VCAM-1+) and “resting” CECs (rCECs, VCAM-1-). Included in these CEC populations are blood vessel-derived endothelial populations (aka endothelial colony forming cells (ECFC) or blood outgrowth endothelial cells (BOEC, CD146+, CD31+, CD45-, CD133-) (16–19). The CEC population also contains bone-marrow derived endothelial progenitor cells (EPCs) that are CD146+, CD31+, CD45-, and CD133+ (20, 21). These endothelial cell populations reflect the activation and health of the vascular endothelium, as illustrated by several recent studies have found that CEC transcription profiles in SCD can distinguish between individuals with stroke (7) or retinopathy (8).

Likewise, in SCD, plasma extracellular vesicles (EVs), comprised of a lipid bilayer and proteins derived from their cell of origin, are increased (22–26). In SCD, numerous reports have found increased EVs derived from multiple cell sources, including red cells, platelets, monocytes, and endothelial cells (5, 22, 23, 25, 27–33). Endothelial cell-derived extracellular vesicles (EEVs) occur from blebbing of the cell membrane in response to activation or apoptosis. EEV production can be stimulated by thrombin as well as cytokines such as interleukin-1 beta (IL-1β) and tumor-necrosis factor-alpha (TNF-α) (34). Likewise, EEVs themselves can activate endothelial cells to generate more EEVs (34). Prior studies, evaluating EEVs from SCD found increased in pro-coagulant tissue factor (TF)-EEVs during periods of crisis, suggesting EEVs may be a valuable indicator of crisis (5).

At present, large-scale SCD clinical trials have excluded direct evaluation of endothelial biomarkers, in part due to pre-analytical and analytical challenges associated with quantitation and assessment of CEC, EEV, and adhesion markers. Herein, we provide a technical report that details and validates isolation and characterization protocols for three surrogate biomarkers of vascular endothelial cell activation within the context of SCD. Additionally, using blood samples from individuals with SCD, we further demonstrate that EEV levels increase during self-reported pain crisis. Overall, we posit that adoption of vascular endothelial cell biomarkers will permit evaluation of therapeutic interventions such as curative gene therapies on endothelial dysfunction.

## Materials and methods

### Chemicals and reagents

Phosphate buffered saline (PBS), Ficoll-Paque PLUS (GE Healthcare), collagen type I, rat-tail tendon (BD Biosciences), Acetic acid solution (Millipore Sigma), heat inactivated fetal bovine serum (Gibco, Invitrogen), EGM-2 BulletKit (Lonza). HT-29 colorectal cells were purchased from ATCC.

### Antibodies

Antibodies were purchased from BioLegend (San Diego, CA). Refer to **Supplementary Table 1** for marker, clone, fluorochrome and catalog information.

### Human blood samples

Blood samples were obtained in sodium citrate, ethylenediaminetetraacetic (EDTA), or sodium heparin Vacutainers^®^ (BD, Columbus, OH, USA) from healthy adult volunteers and individuals with SCD after informed consent and according to protocols approved by the University of Minnesota’s Institutional Review Board in accordance with the Declaration of Helsinki (**Table 1**). For SCD subjects, sample collection was performed in the context of the individual presenting for health care. Clinical variables, including age, gender, sickle cell diagnosis, medications, transfusion history, and pain status were documented in Redcap (Vanderbilt University). To ensure operator blinding, pain events were identified by clinician retrospective review of patient’s chart, which included patient MyChart/emails, administration of pain medications, pain assessment scores from nursing, providers (PA/NP/nurse and physician) assessment, and the context of health care encounter. For healthy controls, samples were collected in research lab and not within health care setting, therefore per IRB, the only variable collected was gender.

**Table 1:** Control and SCD donors’ clinical characteristics.

Clinical parameter	Controls (n=22)	Sickle cell (n=24)
Gender (% female)	63% (n=14)	41% (n=9)
Age (Range)	NA	20-56 (mean 33)
Sickle cell genotype HbSS HbSC HbS-β^+/-^	NA	71% (17) 13% (3) 16% (4)
Hydroxyurea treatment (%)	NA	56%
Average # of ER/Hospitalization or infusion visits in 6 months	NA	2.9 (range 0-11)
Complete Blood Count White blood cells (WBC; 10^7^/dL) Hemoglobin (Hgb; g/dL) Hematocrit (Hct, %)	NA	10.0 ± 2.8 8.8 ± 1.7 25.1 ± 4.6

NA= Not Available as control samples lacking clinical data per IRB stipulations.

### Platelet-free plasma isolation

Blood samples collected in either sodium EDTA or sodium heparin vacutainer tubes were processed down to PFP by performing centrifugation at 2,500x*g* for 15 min at room temperature. The plasma fraction was removed and recentrifuged at 2,500x*g* for 15 min. Aliquots of sample were then made and immediately stored at -80°C.

### Circulating endothelial cell assay

Using 4 mL EDTA whole blood samples, CEC populations were gated and defined as outlined in **Supplementary Figure 1** using the following markers: Total circulating endothelial cells (CECs, CD146+, CD31+, CD45-); Endothelial progenitor cells (EPCs, CD146+, CD31+, CD45-, CD133+); mature CECs (mCECs, CD146+, CD31+, CD45-, CD133-), activated CECs (aCECs, CD146+, CD31+, CD45-, CD133-, CD106+); and resting CECs (rCECs, CD146+, CD31+, CD45-, CD133-, CD106-). Antibodies described in **Supplementary Table 1** were used to identify these CEC subgroups. For enumeration, mononuclear cells were gated using forward and side scatter parameters. For final enumeration, a known concentration of fluorescent counting beads (Invitrogen) was added at the last step before flow cytometry to allow for volume correction in the final calculations.

### Endothelial cell extracellular vesicle isolation and enumeration

EEVs were prepared from heparin PFP by following the International Society on Thrombosis and Haemostasis recommendations (35). Enumeration of plasma EEVs is challenging due to the small size of the particles. The lower limit of detection of EEVs using a standard flow cytometer is typically 0.2 µm. To standardize our detection, we used commercially available fluorescent size beads (0.1 – 2.0 µm diameter) to set the proper size gates for data collection; for our analysis, a 0.2 – 1.0 µm gate was used for enumeration (**Supplementary Figure 2**). EEV populations were gated and defined as outlined in **Supplementary Figure 3** to identify the EEV subgroups.

### Optimization of antibody concentrations used for CEC and EEV flow cytometry

Each primary antibody was titrated using flow cytometry to determine optimal antibody concentrations for CEC and EEV identification (**Supplementary Figure 4**). The optimal monoclonal antibody concentrations were determined by adding increasing primary antibody concentrations to a mixture of cells that were positive and negative for the antigen of interest. Separation Index (SI) is used to calculate the difference in signal between the positive and negative populations while taking the spread of the negative into account. (36) The optimal antibody concentration can be found where the separation index plateaus. All values in the formula were calculated using FloJo software (BD Biosciences).


$$\text{SI}=\frac{\text{median signal}-\text{median background}}{\left[\left(84\text{th percentile median background}-\text{median background}\right)/0.995\right]}$$

### CD146 and CD31 optimization using SI

FITC-labeled anti-CD146 antibody SI was determined using blood outgrowth endothelial cells (BOEC, CD146+) and HT-29 adenocarcinoma cells (CD146-). The optimal CD146 antibody concentration was 0.1 µg/100 µl (**Supplementary Figure 4A**).

CD31 optimization. BV421-labeled anti-CD31 antibody SI was determined using blood BOEC (CD31+) and HT-29 cells (CD31-). Optimal CD31 antibody concentration was 0.25 µg/100 µl (**Supplementary Figure 4B**).

CD106, CD133, and CD45 optimizations were completed in similar manner using appropriate positive and negative controls (data not shown). For CD106, the optimal antibody concentration was 0.2 µg/100 µl. For CD133, the optimal antibody concentration was 1.0 µg/100 µl. For CD45, the optimal antibody concentration was 0.25 µg/100 µl.

### Soluble adhesion molecule assays

Soluble adhesion molecules were measured in EDTA PFP using a LegendPlex multiplex assay following the manufacturer’s directions (BioLegend). Initial studies comparing controls verse subjects with SCD using the LEGENDplex™ human adhesion molecule (#740946) (**Table 2**). Due to shortages of the materials to make the panel during COVID, a change in materials was made; the remainder of the analysis was completed using LEGENDplex™ Custom Human 4- Plex panel (90000637, lot B33057) (**Table 3**). EDTA was selected as anticoagulant for analysis as endothelial cell P-selectin cannot be accurately measured in serum due to platelet degranulation and release of platelet P-selectin into serum upon clotting.

**Table 2:** Soluble adhesion molecules in control verses SCD subjects.

Soluble adhesion molecule	Control n=11	SCD n=19	p-value
E-Selectin (ng/mL)	5.8 ± 2.2	12.3 ± 2.5	*0.005
P-selectin (ng/mL)	1.4 ± 0.2	2.9 ± 0.5	*0.012
VCAM-1 (ng/mL)	49.2 ± 9.6	98.5 ± 15.4	*0.009
ICAM-3 (ng/mL)	22.5 ± 4.8	63.9 ± 18.1	*0.002

All samples run on LEGENDplex™ human adhesion molecule panel.

Mean ± SEM with significance (*) determined by Mann-Whitney test.

**Table 3:** Soluble adhesion molecules in SCD subjects based on steady state verses crisis status.

Soluble adhesion molecule	No crisis n=9-13	Crisis n=14-16	p-value
E-Selectin (ng/mL)	20.8± 5.8 (n=13)	15.5 ± 5.3	0.5
P-selectin (ng/mL)	16 ± 13 (n=10)	3.2 ± 0.6	0.8
VCAM-1 (ng/mL)	85.5 ± 15.4 (n=9)	141.7 ± 41.6	0.3
ICAM-3 (ng/mL)	68.7 ± 27.9 (n=10	58.3.4 ± 13.1	0.7

All samples run on LEGENDplex™ Custom Human 4- Plex panel.

Mean ± SEM with significance (*) determined by Mann-Whitney test.

### Statistics

Descriptive statistics are presented as means ± standard error means (SEM) as indicated. Normality assessments were conducted prior to comparisons with Mann Whitney t-testing used for non-normal population and t-test with Welch correction used for normal populations using GraphPad Prism (v 9).

## Results

### Soluble adhesion molecules are increased in SCD

To determine if endothelial activation is present in SCD individuals, we first measured soluble adhesion molecules. Compared to control platelet-free plasma, plasma from SCD individuals exhibited significantly increased sE-selectin, sP-selectin, sVCAM-1, and sICAM-3 (**Table 2** **).**

Next, we evaluated if soluble endothelial markers changed based on patient-report pain crisis. Clinical characteristics from the electronic medical records are presented in **Table 1**. Vaso-occlusive crises were either self-reported or documented during visits to the clinic, infusion center or hospital. Data were collected over a period of 20 months (Nov 2019-June 2021), with sample collection interrupted from March 2020-Nov 2020 due to COVID-19 restrictions, blood samples from consented individuals with SCD were collected from M Health Fairview. Consistent with previous publications comparing soluble endothelial marker data in SCD subjects at baseline steady state to pain crisis (37–39), we found no difference in soluble adhesion molecules between individuals at steady state versus crisis (**Table 3** **).**

### CEC and EEV assay validation

Validation and description of antibodies and methods used in the CEC and EEV assays are described in the **Supplementary Methods** and **Supplementary Figures 5**-**11** in the **Supplementary Material**.

### CEC numbers in blood are increased in SCD

To determine if the circulating endothelial cells were increased in SCD individuals, we measured total CEC and CEC subpopulations in control and SCD subjects. Compared to controls, SCD individuals had a significantly higher (p<0.05) number of total CECs, mCECs, and rCECs in whole blood (**Table 4**). There was not a significant difference in other CEC subpopulations, including activated CECs. Due to the need to perform the CEC assays on fresh samples, there were a limited number of samples with CEC measurement. Compared to SCD patients in steady state, SCD patients in pain crisis did not have significant differences in total CECs or in CEC subpopulations (**Table 5**). We were not able to assess longitudinal CEC levels in any subject. Overall, our limited data suggest that CEC levels alone are not sufficient to reflect acute vaso-occlusion, however additional longitudinal data following this marker over time in SCD individuals potentially might better reflect overall changes in vascular activation.

**Table 4:** Circulating endothelial cell populations (CECs) in control versus SCD subjects.

CEC population Per mL blood	Control n=21	SCD n=21	p-value
Total circulating endothelial cells CD146+, CD31+, CD45-	46.0 ± 6.3	104 ± 17.1	*0.003
Endothelial progenitor cells EPCs: CD146+, CD31+, CD45-, CD133+	8.3 ± 2.8	18.8 ± 4.7	0.91
Mature circulating endothelial cells mCECs: CD146+, CD31+, CD45-, CD133-	37.7 ± 5.4	84.8 ± 16.1	*0.008
Activated circulating endothelial cells aCECs: CD146+, CD31+, CD45-, CD133-, CD106+	20.6 ± 4.8	27.7 ± 7.7	0.57
Resting circulating endothelial cells rCEC: CD146+, CD31+, CD45-, CD133-, CD106-	17.1 ± 4.1	56.8 ± 13.4	*0.007

Values mean ± SEM with *significance determined by Mann-Whitney test.

**Table 5:** Circulating endothelial cell populations (CECs) in SCD subjects based on steady-state verses crisis status.

CEC population Per mL blood	Steady state n=7	Crisis n=13	p-value
Total circulating endothelial cells CD146+, CD31+, CD45-	105.51 ± 31	102.3 ± 82.3	>0.999
Endothelial progenitor cells EPCs: CD146+, CD31+, CD45-, CD133+	14.3 ± 7.5	18.1 ± 20.4	0.92
Mature circulating endothelial cells mCECs: CD146+, CD31+, CD45-, CD133-	91.0 ± 26.5	83.7 ± 80.8	0.64
Activated circulating endothelial cells aCECs: CD146+, CD31+, CD45-, CD133-, CD106+	37.0 ± 17.8	23.5 ± 30.2	0.82
Resting circulating endothelial cells rCEC: CD146+, CD31+, CD45-, CD133-, CD106-	54.0 ± 15.4	52.5 ± 73.0	0.44

Mean ± SEM.

### Activated EEV numbers in plasma are increased in SCD

As activated endothelial cells can shed EVs, we next compared EEVs in controls and SCD individuals. Activated EEVs (aEEVs) were significantly higher (p=0.0017) in the blood of SCD subjects as compared to controls (**Table 6**). Combined with the soluble adhesion molecule and CEC data, the EEV data further confirms that individuals with SCD have increased vascular activation.

**Table 6:** Endothelial-derived extracellular vesicle (EEV) populations EVs in control verses SCD subjects.

EEV population x 10,000/mL platelet free plasma	Control n=16	SCD n=16	p-value
Total endothelial extracellular vesicles (Total EEV) CD146+, CD31+, CD45-	8.4 ± 3.4	46.9 ± 23.4	0.1100
Endothelial progenitor vesicle EPV: CD146+, CD31+, CD45-, CD133+	6.3 ± 3.1	38.1 ± 21.1	0.1713
Mature endothelial extracellular vesicle mEEV: CD146+, CD31+, CD45-, CD133-	2.0 ± 0.5	7.5 ± 3.6	0.2065
Activated endothelial extracellular vesicle aEEV: CD146+, CD31+, CD45-, CD133-, CD106+	0.029 ± 0.023	1.1 ± 0.7	*0.0017
Resting endothelial extracellular vesicle rEEV: CD146+, CD31+, CD45-, CD133-, CD106-	1.7 ± 0.5	6.4 ± 3.2	0.2099

Mean ± SEM with significance (*) determined by Mann-Whitney test.

### EEV numbers in plasma are higher in SCD individuals reporting vaso-occlusive crisis

Unlike soluble adhesion markers, previous studies evaluating EEVs and pain crisis have reported some modest correlation between episodes of acute pain crisis and increased EEVs (5, 40). Therefore, we evaluated if plasma-based EEV increased in our SCD subjects reporting pain crisis. Compared to SCD patients in steady state, SCD individuals in pain crisis exhibited an increase in the total number of EEVs and activated EEVs (**Table 7**). Collectively, these data suggest that overall EEVs, but not adhesion molecules, may correlate with patient-reported pain crisis.

**Table 7:** Endothelial-derived extracellular vesicle (EEV) populations in SCD subjects based on steady state verses crisis status.

EEV Population x 10,000/mL platelet free plasma	Steady state n=3	Crisis n=9	p-value
Total endothelial extracellular vesicles (Total EEV) CD146+, CD31+, CD45-	1.1 ± 0.4	13.9 ± 5.3	*0.04
Endothelial progenitor vesicle EPV: CD146+, CD31+, CD45-, CD133+	0.15 ± 0.08	15.9 ± 7.5	0.16
Mature endothelial extracellular vesicle mEEV: CD146+, CD31+, CD45-, CD133-	1.85 ± 1.3	1.9 ± 0.6	0.7
Activated endothelial extracellular vesicle aEEV: CD146+, CD31+, CD45-, CD133-, CD106+	0.02 ± 0.02	0.2 ± 0.08	*0.04
Resting endothelial extracellular vesicle rEEV: CD146+, CD31+, CD45-, CD133-, CD106-	2.5 ± 1.2	1.5 ± 0.5	0.7

Mean +/- SEM with significance (*) determined by t-test with Welch correction.

## Discussion

Endothelial dysfunction is a hallmark of many inflammatory and thrombotic diseases, including SCD. Our study provides validated methods for laboratory analysis of CECs, EEVs, and soluble adhesion molecules. We identify optimal pre-analytic variables, including sample anticoagulation, antibody titration, and storage. In individuals with SCD, total CECs, mCECs, rCECs, aEEVs, and soluble adhesion molecules were increased as compared to healthy control subjects. Furthermore, during SCD pain crisis, total EEVs and aEEVs were increased as compared to SCD individuals in steady-state. Collectively, our study provides methods by which the research community can incorporate endothelial activation markers into clinical trials, specifically adhesion markers, CECs, and FEEVs.

After years of SCD research, the last decade has seen an unprecedented advancement of multiple potential therapeutics to prevent hemolysis and reduce vascular activation. Furthermore, development of definitive therapies for SCD based on genetic manipulation of sickle hematopoietic stem cells (HSCs) constitute a transformative advance and possible cure. The delivery of anti-sickling genes into long-term repopulating HSCs allows the production of corrected red cells that will not hemolyze and therefore ameliorate the anemia, prevent vaso-occlusion, interrupt I/R pathophysiology to reduce inflammation and vascular cell activation. However, given the only recent approval of these agents, it is unknown if subjects with SCD will exhibit a reduction in chronic vascular issues.

Overall, the advancement and development of theses numerous disease-modifying therapies, including curative therapies, has increased the need for strategies to stratify/advise SCD patients toward the therapies most likely to reduce and prevent complications. Because endothelial dysfunction contributes to several major SCD-related complications, including thromboembolism, stroke, and pulmonary hypertension, we chose to evaluate three separate, but related vascular activation markers in SCD. Our aim was to provide framework for method harmonization and assist with identifying potential correlative biomarkers. Of note, in a similar fashion, a recent study, known as the ELIPSIS study, monitored patients with SCD at home with an electronic patient-reported outcome tool and actigraphy over the course of 6 months. When VOE was reported, a mobile-based blood collection team obtained blood samples, which permitted investigators to analyze trends over the course of a patient-reported VOE (41). During times of patient-reported VOE at home, whole blood obtained from SCD subjects exhibited significant increased adhesion to P-selectin when compared to the same SCD subject at baseline/steady state (41). This data suggests that a functional marker of vascular activation, such as P-selection adhesion, may correlate with patient-reported VOC. Of note, in our studies, two subjects did start anti-P-selectin therapy with crizanlizumab during study collection. We noted that consistent with mechanisms of activation, soluble P-selectin levels in these subjects were inhibited (data not shown).

CEC measurement to discern vascular activation is of interest in several other disorders, including stem cell transplant where sinusoidal obstruction syndrome (SOS), otherwise known as veno-occlusive disease (VOD) can occur. In SOS/VOD, endothelial activation is known to contribute to pathogenesis, with several markers, such as VCAM-1, increased in subjects with the disease. A recent study sought to assess CEC levels in stem cell transplant subjects using CellSearch, a semi-automated system. The authors found that compared to individuals with lower CEC count, those with a higher percentage of CECs were likely to exhibit SOS/VOD (hazard ratio 12.9, confidence interval 1.2-138.2) (42). Our studies did not use the CellSearch system, however, availability of this device, or development of harmonized flow cytometry protocols such as ours, may permit dissemination of CEC measurement in trials or outpatient monitoring. The correlation between CEC and SOS/VOD severity is notable as in SCD children who undergo HSC transplant, the overall incidence of SOS/VOD is as high as 30% (43). Therefore, moving forward, as several recently or soon-to be approved SCD curative strategies include allo- or autologous transplant with conditioning, information regarding baseline, pre- and post-transplant CEC measurements in individuals with SCD may be useful in predicting which subjects may need preventative treatment.

Along with our present study, multiple other groups have observed increased number of EVs in SCD individuals (5, 22, 23, 26–28, 40). In particular, in SCD red-cell derived EVs (RBC-EV) have been found to deliver heme to endothelial cells which results in activation, coagulation, and ultimately vaso-occlusion (25, 31, 44). Interestingly, treatment of SCD subjects with HU treatment, the current gold standard therapy, decreased RBC-EV stimulation of endothelial cells (23). This suggests that EV measurement is sensitive to therapy-related changes in vascular activation. In our study we found that use of heparin vacutainers increased yield of endothelial-derived EVs. In general, many EV studies avoid heparin anticoagulated samples as heparin blocks uptake of EVs into recipient cells (45, 46). Of note, other groups have conducted extensive pre-analytic measurements of EVs and found that in general, the period of time between isolation, centrifugation procedure (single or double-spun), and storage temperature were the main drivers of variability (47). Interestingly, in pulmonary hypertension subjects, EEVs measured in sodium citrate decreased with pulmonary rehab (48). Additionally, recent studies using EEVs isolated from obese subjects to treat cardiac myocytes found increased markers of fibrosis (49). As pulmonary hypertension is a major driver of morbidity in SCD, one could posit that longitudinal measurement over time of EEVs may help reflect subjects at risk for this complication.

CD106 (VCAM-1) was used to discern activated from resting EEVs and CECs in our studies. CD106 can be internalized, expressed on cell surface, or shed depending on the environment of the cell. Circulating endothelial cells are non-hematologic cells with nature endothelial identity and limited growth capacity. It is plausible that during circulation CECs internalize VCAM-1 or, through interactions with neutrophil elastase or cathepsin G, shed CD106 into the circulation. EVs, which are smaller and released by specific triggers, contain pieces of cell membrane and are not able to internalize VCAM-1. This may account for differences between aCEC and aEEVs observed.

Our study has several limitations. First, in our method we were unable to enumerate EVs smaller than 0.2 µm, such as exosomes. Similarly, we did not enumerate EVs larger than 1.0 µm. Our recovery rate of EVs was 80-90% in our spike-recovery experiments (**Supplementary Figure 9C**) suggesting that 10-20% of EVs were less than 0.2 µm or larger than 1.0 µm. Therefore, further studies to evaluate these smaller (or larger) particles may be warranted. Second, due to the timing of the sample collection, COVID-19 research restrictions limited and reduced subject recruitment. This prevented sufficient samples to discern if endothelial biomarkers vary due to SCD genotypes; i.e., do HbSC subjects have different endothelial activation patterns compared to HbSS? Furthermore, several subjects started two new medications, crizanlizumab and voxelotor, during the study period; due to small number, our studies are under powered to determine if these medications changed our markers. Additionally, due to ~9 month period where we were unable to obtain samples, we were unable to fully complete paired-sample analysis to characterize patients before, during, and after VOC. Additionally, in part due to studies evaluating COVID-19 coagulopathy, multiple reagents used in our studies were consistently in low supply. Therefore, we had to change reagents for adhesion studies. Last, we were unable to implement multi-center analyses or ascertain if shipping samples overnight would match our simulations. Further studies to address these pitfalls are warranted. Finally, a major limitation unrelated to COVID remains; both CEC and EEV enumeration require flow cytometry, which adds both time and training related challenges. Therefore, further harmonization studies and regular inter-center assessment of precision and accuracy are required (50).

In conclusion, we report that in SCD flow cytometry-based analytic assessment of adhesion molecules, CECs, and EEVs offer an accurate, precise and reproducible measure of endothelial activation. Collectively, we propose that in SCD individuals these markers may provide non-invasive biomarkers for endothelial injury. Overall, we report that addition of heparin vacutainers to blood collection allows for improved EEV enumeration. Additionally, we demonstrate that both soluble adhesion molecules and EEV quantification are amenable to central analysis with sample freezing conducted prior to shipping. In contrast, CEC enumeration requires assessment in fresh blood samples whenever possible. Our studies suggest that EEV elevation may correlate with patient-reported incidence of crisis. Further standardization of endothelial activation measurements across trials in individuals with SCD may provide an up-to-the-minute snapshot of the health of the vessel wall.

## Data Availability

The original contributions presented in the study are included in the article/**Supplementary Material**. Further inquiries can be directed to the corresponding author.
