# Supplementary material for: Missing the mark(ers): circulating endothelial cells and endothelial-derived extracellular vesicles are elevated in sickle cell disease plasma
Source: Front Immunol. 2024 Dec 24;15:1493904. doi: 10.3389/fimmu.2024.1493904 (PMC11703723; doi:10.3389/fimmu.2024.1493904)
Supplement: Supplementary file 1 [file DataSheet1.pdf]

SUPPLEMENTAL MATERIAL

Supplemental Methods

Validation of primary antibodies used for CEC and EEV identification

Monoclonal antibody specificity is defined by how well the antibodies recognize the antigenic target without cross- reacting with off-target antigens. Manufacturers are responsible for reagents having the correct specificity listed on their labels. We further validated primary antibody specificity for CEC and EEV assays using various endothelial cells in culture and immunofluorescence (IF) confocal microscopy. We first used IF for MCAM-1 (CD146), PECAM-1 (CD31) and VCAM-1 (CD106) in human blood outgrowth endothelial cells (BOEC) and EEVs in conditioned media collected from BOEC. BOEC obtained from peripheral blood can be expanded to vast numbers and are amenable to cryopreservation. We used an established repository of cryopreserved BOEC from both healthy and sickle African Americans.[1,2] Primary human umbilical vein endothelial cells (HUVEC) were isolated from human umbilical cords as previously described.[3] All cells were grown until 95% confluent and used between passages 2-8. Both BOEC and HUVEC were used to validate sensitivity and specificity of CEC and EEV assays and evaluate pertinent pre-analytical variables including processing time, storage temperature, and collection methods.

After thawing and expansion, BOEC were immunostained with primary monoclonal antibodies to endothelial cell markers CD146, CD31, CD106, or an isotype control followed by the appropriate fluorescence-labeled secondary antibody. BOEC expressed CD146 and CD31 (**Supplemental Figure 5**). For CD106 staining, BOEC were pre-treated for 4 hr with or without TNF-α (10 ng/ml), an activator of CD106 expression. Untreated BOEC had little to no CD106 expression, but BOEC treated with TNF-α had both CD146 (green) and CD106 (red) expression. CD146 and CD106 were often co-localized (yellow) on BOEC.

Conditioned media was collected from BOEC cells after 72 hr in culture. Media was centrifuged at 300 x g to remove debris, EEVs were purified from the media using a Capturem™ spin column containing a lectin-based binding compound to capture the EEVs after removal of large cellular debris (Takara). Purified EEVs were dried onto a glass slide followed by immunofluorescent staining. BOEC-derived EEVs expressed CD146 and CD31 (**Supplemental Figure 6,** upper

panel). BOEC were treated with TNF-α (20 ng/ml) for 12 hr then BOEC-conditioned media was collected 72 hr later for EEV isolation and immunofluorescent staining with anti-CD31 (PECAM-1) and CD106 (VCAM-1) EEVs isolated from BOEC-conditioned media after 12 hr TNF- α treatment, expressed CD31 and CD106 (**Supplemental Figure 6,** lower panel).

Leukocyte-specific antigen (CD45) is a marker for hematopoietic cells and was used as a negative selection marker for CECs and EEVs. Peripheral blood mononuclear cells were isolated on a Ficoll-Paque gradient, washed, and centrifuged onto glass cover slips. Mononuclear cells were stained for CD45 followed by staining with the appropriate FITC (green)-labeled secondary antibody (**Supplemental Figure 7,** right upper panel). Control mononuclear cells were incubated with an isotype control antibody followed by the same FITC-labeled secondary antibody to the CD45 antibody. BOEC (CD45-, VE-cadherin+) were stained with antibodies to CD45 and VE-cadherin.

Next, to validate CD45 antibody staining in EVs, platelet-derived EVs were purified from human plasma using a Capturem^{TM} spin column. Purified EVs were dried onto a glass slide and stained for CD45 and CD41 (platelet-specific marker) **(Supplemental Figure 8,** lower panel).

Prominin-1 (CD133) is a marker for endothelial progenitor cells (EPCs). However, BOEC do not express CD133. To validate the CD133 antibody, we used a colorectal carcinoma cell line, HT-29 that expresses CD133.[4] The anti-CD133 antibody avidly stained HT-29 cells (**Supplemental Figure 8,** upper panel). The antibody did not stain BOEC (data not shown). Likewise, EVs from HT-29-conditioned media were collected and purified using a Capturem^{TM} spin column. Purified EMIVs were dried onto a glass slide and stained with the primary anti-CD133 antibody followed by the appropriate PE (red)-labeled secondary antibody (**Supplemental Figure 8,** lower panel**).**

Dynamic range and analytic precision of CEC and EEV flow cytometry assays

As CECs are a rare cell population, we sought to validate the dynamic range and analytic precision for our protocol using BOEC as authentic CECs. Previous work by our group established a repository of expanded and cryopreserved BOEC isolated from the peripheral blood of healthy and sickle African Americans.[1,2] Therefore, we performed spike-recovery (i.e. “add-back”) experiments of known numbers of BOEC to whole blood and EEVs derived from BOEC to PFP to elucidate the dynamic range of detection for our CEC and EEV flow cytometry assays (**Supplemental Figure 9A**). For our CEC assay, increasing numbers of BOEC (n=4/group) were

added to healthy control blood. Cells were then stained and analyzed by flow cytometry to enumerate total CECs ($CD146^+$, $CD31^+$, $CD45^-$). In **Supplemental Figure 9B**, the measured number of CECs were plotted against the expected number of CECs. Our CEC assay was linear between 0 and 4500 CECs ($r^2 = 0.9977$) with a recovery rate of approximately 95%.

For validation of the EEV assay, EEVs were isolated from BOEC-conditioned media after 72 hours using a lectin-based Capturem^{TM} EV isolation kit (Takara). Purified EEVs were enumerated using a NanoSight LM-10 nanoparticle tracking analyzer (NTA). Purified BOEC EEVs were added back to PFP to assess the dynamic range of EEV enumeration by flow cytometry. **Supplemental Figure 9C** demonstrates the recovered number of EEVs ($CD146^+$, $CD45^-$) against the expected number of EEVs; the assay was linear between ~500 and 160,000 EEVs ($r^2 = 0.9995$), with a recovery rate (measured/expected %) of 80 - 90%.

To determine the analytical precision of our assays, we repeated CEC enumeration 4-6 times from SCD blood samples (n=5). The average CV (SD/mean %) ranged from 5.7% to 22.6% (**Supplemental Table 2**). Similarly, for EEVs, we repeated EEV enumeration 5-7 times from the same PFP samples (n=5). The average CV (SD/mean %) ranged from 9.7% to 13.2% (**Supplemental Table 3**).

Verification of pre-analytical sample collection and storage parameters for CEC, EEV and soluble adhesion molecules

In large clinical studies, standardization of methods across study locations and/or centralized laboratory analysis is desirable. Therefore, we sought to establish and optimize blood collection and storage parameters for enumerating CECs and EEVs. Prior work has established that CEC isolation and enumeration should occur immediately after blood collection to avoid a slow, but steady, decline in CEC population.[5] To confirm previous findings and to mimic sample shipping conditions that might be used in a clinical trial, we compared CEC analysis in fresh blood to samples stored for 24 hr at 4ºC or at room temperature (22 ºC, RT). Confirming previous studies, there was a sharp decrease in the number of total, mature, activated, and resting CECs in blood stored at 4ºC for 24 hr (**Supplemental Figure 10A**). Interestingly, in blood samples stored at RT (22^0C) for 24 hr, there was a much better agreement with freshly isolated samples (**Supplemental Figure 10B**). Overall, our data support previous work, but also suggest that if needed, 24 hr shipping at room temperature may permit centralized CEC enumeration.

Prior work has reported that EEV values are sensitive to the type of anticoagulant used in blood collection.[6] Historically, clinical blood samples are collected in either citrate or EDTA anticoagulants, which may decrease or eliminate EEVs due to adhesion of the endothelial-derived EVs to platelets or other formed elements.[6,7] Others have suggested that protease inhibitor anticoagulants, including heparin, preserve EEV counts. Therefore, to ascertain which collection tube would be preferred for EEV enumeration, we compared EEV enumeration in EDTA and heparin PFP. Compared to EDTA collected samples, heparin collected PFP samples exhibited increased total EEVs, EPCs, mEEVs, aEEVs, and rEEVs (**Supplemental figure 11**). Indeed, in PFP isolated from blood collected in EDTA, detection of EEVs was low, suggesting that for optimal EEV isolation, heparin vacutainers are ideal.

SUPPLEMENTAL TABLES

Supplemental Table 1: Antibodies used to define CECs and EEVs

CD Marker	Common Name	Marker	mCECs mEEVs	rCECs rEEVs	aCECs aEEVs	EPCs pEEVs	Clone	Fluoro-chrome	Biolegend Catalog #
CD31	PECAM-1	Endothelial Cells	+	+	+	+	WM59	BV421	303123
CD146	MCAM	Endothelial Cells	+	+	+	+	P1H12	FITC	361011
CD45	PTPRC	Hematopoietic Cells	-	-	-	-	2D1	APC/Cy 7	368515
CD133	Prominin-1	Endothelial Progenitor Cells	-	-	-	+	7	APC	372805
CD106	VCAM-1	Activated Endothelial Cells	±	-	+	±	STA	PE	305805

Supplemental Table 2: CEC assay precision

CEC	Patients (n)	Replicates (n)	CV (%)
mCEC	5	4-6	5.7
aCEC	5	4-6	19.5
rCEC	5	4-6	6.3
EPC	5	4-6	22.6

Supplemental Table 3: EEV assay precision

EEV	Patients (n)	Replicates (n)	CV (%)
Total EEV	5	5-7	9.7
mEEV	5	5-7	10.3
rEEV	5	5-7	13.2

SUPPLEMENTAL FIGURES

Supplemental Figure 1

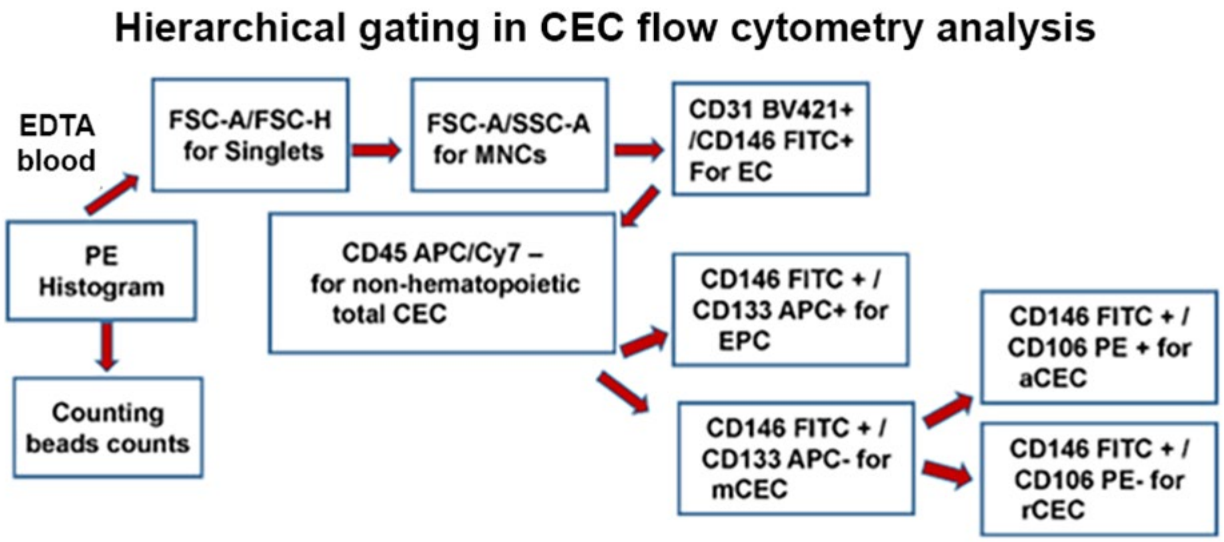

Supplemental Figure 1. Hierarchical gating for CEC flow cytometry analysis. A known concentration of PE fluorescent counting beads was added to each blood sample so that CEC enumeration could be accurately determined per mL of blood. For CEC gating, the first gates applied were to identify singlet cells using forward scatter area (FSC-A) and forward scatter height (FSC-H) gating and to identify mononuclear cells (MNCs) using FSC-A and SSC-A gating. Then CECs were identified (CD31+/CD146+) as non-hematopoietic (CD45-). Next, endothelial progenitor cells (EPCs, CD133+) and mature CECs (mCECs, CD133-) were identified. The mCECs were further subdivided into activated CECs (aCECs, CD106+) and resting CECs (rCECs, CD106-).

Supplemental Figure 2

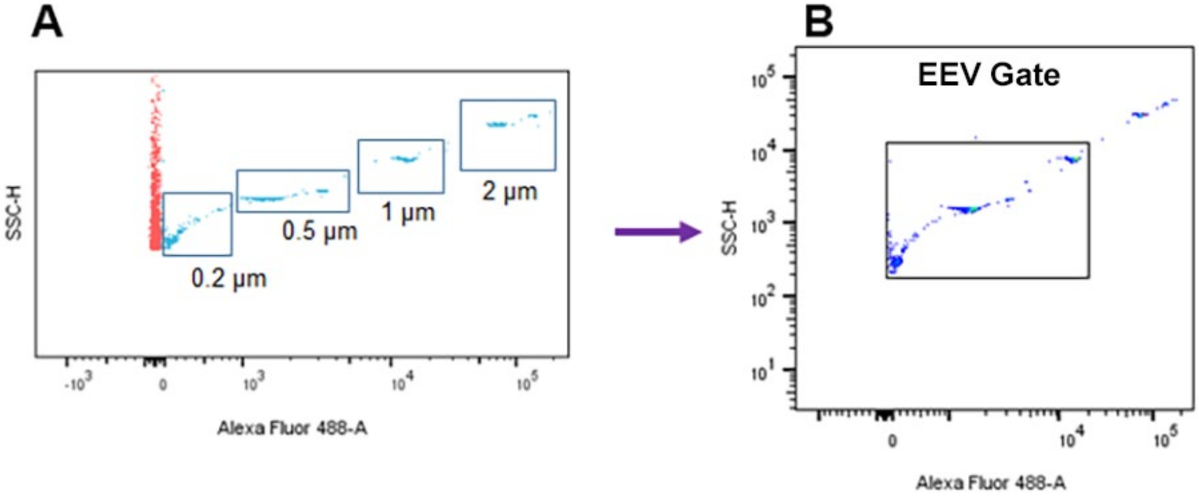

Supplemental Figure 2. EEV gating protocol using fluorescence size beads. (A) Fluorescent size beads (0.2, 0.5, 1.0, and 2.0 µm diameter) labeled with Alexa Fluor 488 were used to set the proper flow cytometry size gates for EEVs (left panel). The beads have an increasing amount of fluorescence as their diameter increases. Cytometer noise can be seen in the side scatter channel (SSC-H) to the left of the 0.2 µm size gate. (B) A 0.2 – 1.0 µm size gate was used for EEV analyses so that only signals within that size range were enumerated.

Supplemental Figure 3.

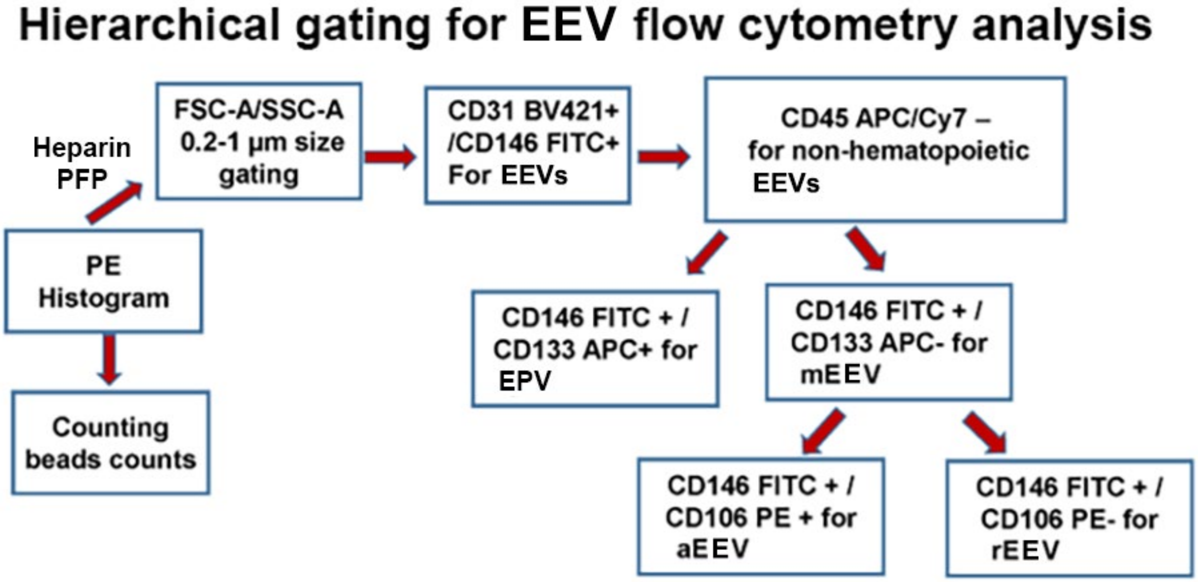

Supplemental Figure 3. Hierarchical gating for EEV flow cytometry analysis. A known concentration of PE fluorescent counting beads was added to each heparin platelet-free plasma (PFP) sample so that EEV enumeration could be accurately determined per mL of PFP. For EEV gating, the first gate applied was the size gate (0.2 – 1.0 µm diameter, see Supplemental Figure 2) followed by gating for endothelial cells (CD31+/CD146+) that were non-hematopoietic (CD45-). Next, endothelial progenitor EV (EPV, CD133+) and mature EEV (mEEVs CD133-) were identified. The mEEVs were further subdivided into activated EEV (aEEV, CD106+) and resting EEV (rEEV, CD106-).

Supplemental Figure 4

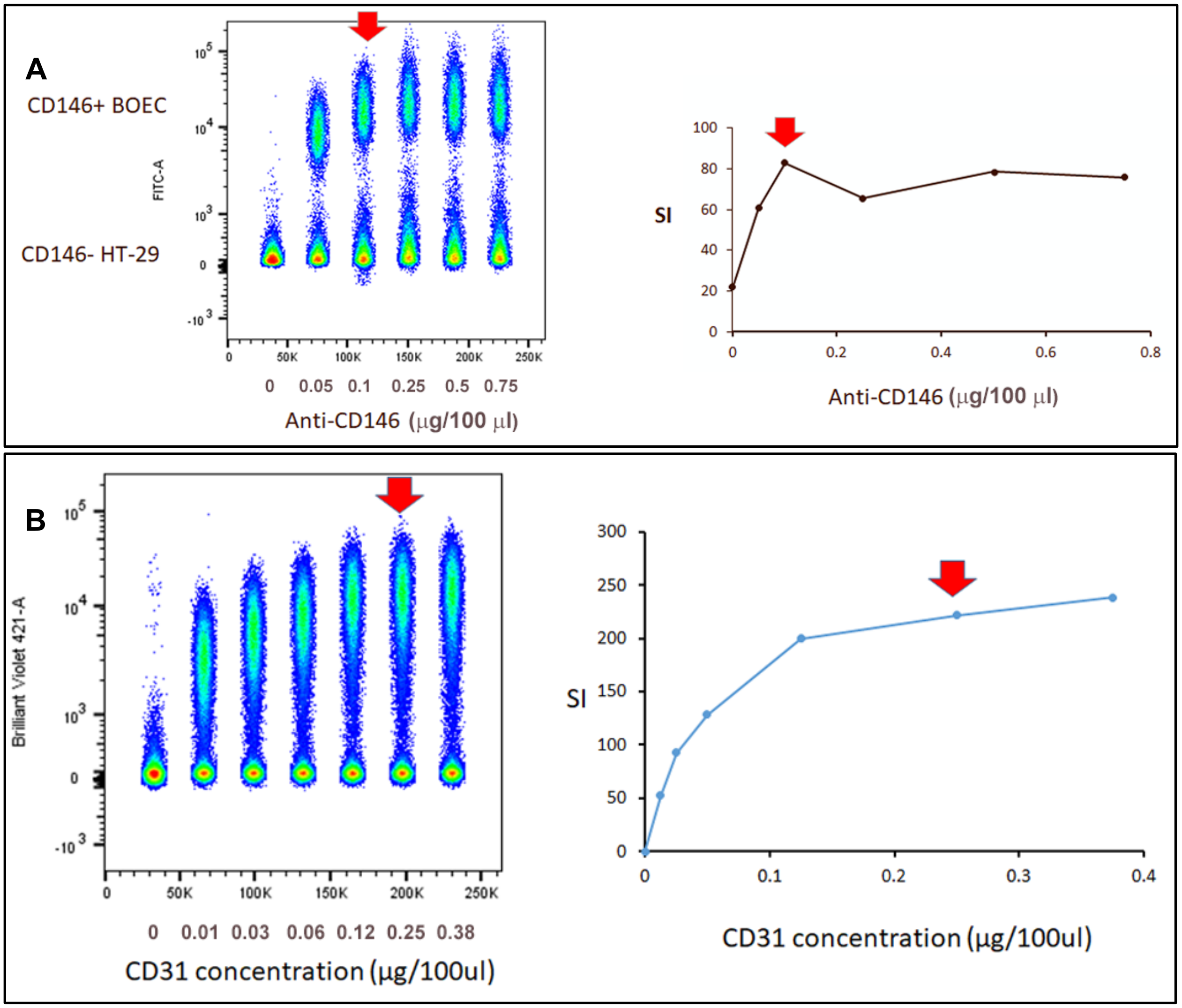

Supplemental Figure 4: Optimization of antibodies for CEC and EEV detection using flow cytometry. (A) BOEC (CD146+) and HT-29 adenocarcinoma cells (CD146-) (0.5 – 1.0 million cells each) were stained with increasing concentrations (0 – 0.75 µg/100 µl) of FITC-labeled anti-CD146 antibody. (B) BOEC (CD31+) and HT-29 cells (CD31-) (0.5 – 1.0 million cells each) were stained with increasing concentrations (0 – 0.38 µg/100 µl) of BV421-labeled anti-CD31 antibody. For both panels, the separation index (SI) was calculated using FloJo software with the red arrows indicating the optimal anti-CD146 and anti-CD31 antibody concentrations.

Supplemental Figure 5.

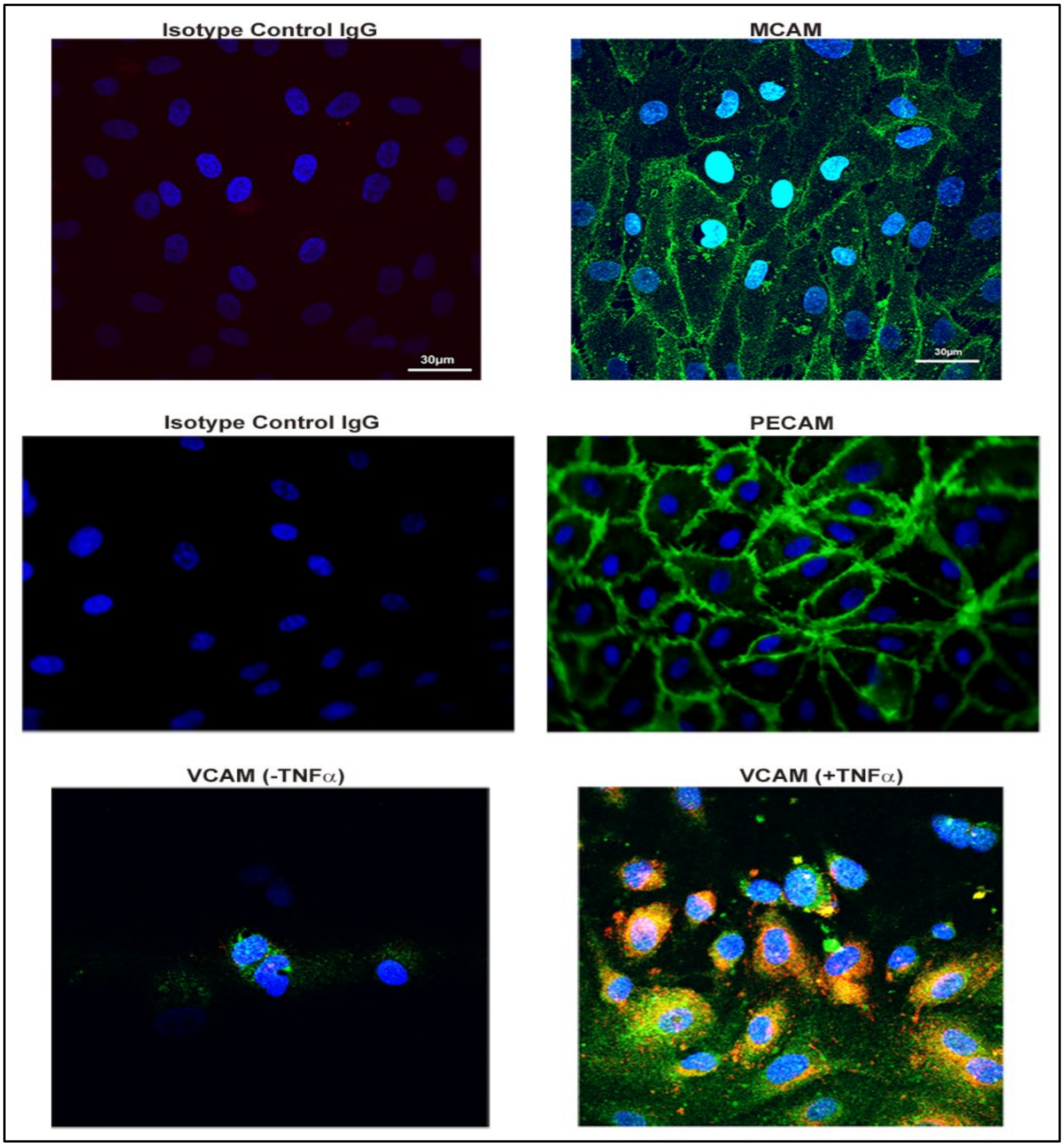

Supplemental Figure 5: Validation of CEC antibodies. Blood outgrowth endothelial cells (BOEC) were cultured until confluent followed by immunofluorescence staining for MCAM-1 (CD146), PECAM-1 (CD31), VCAM-1 (CD106), or isotype control IgG. Nuclei were stained blue with DAPI. MCAM-1 and PECAM-1 were stained with FITC (green)-labeled and VCAM-1 was stained with PE (red)-labeled secondary antibodies. For VCAM-1 staining, BOEC were pre-treated with TNF-α (20 ng/ml) for 4 hr to induce VCAM-1 expression and stained with primary antibodies to VCAM-1 and MCAM-1. Images were collected using fluorescence confocal microscopy at 60X.

Supplemental Figure 6

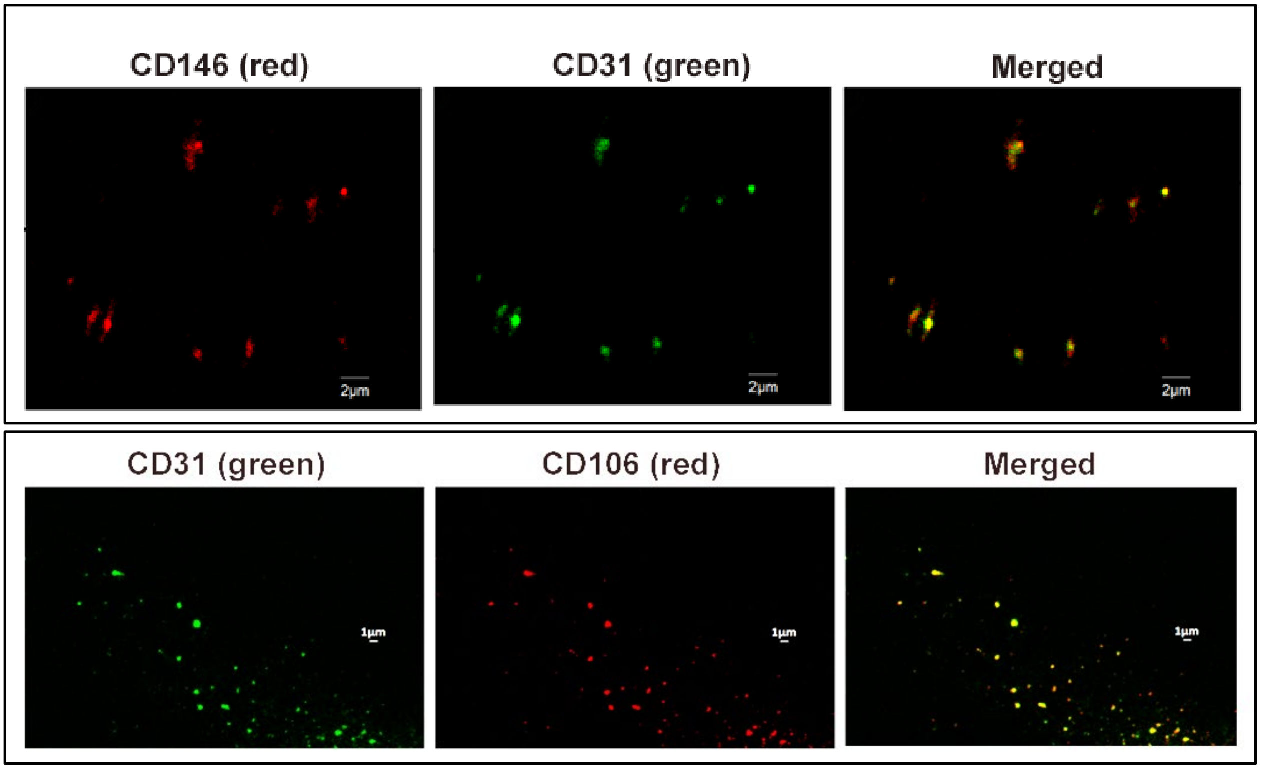

Supplemental Figure 6: Validation of EEV antibodies. BOEC-conditioned media was collected after 72 hr and EEVs were isolated from the media using a Capturem^{TM} spin column. Purified EEVs were dried onto a glass slide followed by immunofluorescent staining using anti-CD146 (MCAM-1) and CD31 (PECAM-1) followed by the appropriate FITC (green)-labeled (CD31) or PE (red)-labeled (CD146) secondary antibodies. Images were collected using fluorescence confocal microscopy at 120X. CD146 and CD31 images were overlayed in the merged image. Lower panel: BOEC were treated with TNF-α (20 ng/ml) for 12 hr then BOEC-conditioned media was collected 72 hr later for EEV isolation and immunofluorescent staining with anti-CD31 and CD106 (VCAM-1) followed by the appropriate FITC (green)-labeled (CD31) or PE (red)-labeled (CD106) secondary antibodies. Images were collected using fluorescence confocal microscopy at 120X. CD31 and CD106 images were overlayed in the merged image.

Supplemental Figure 7

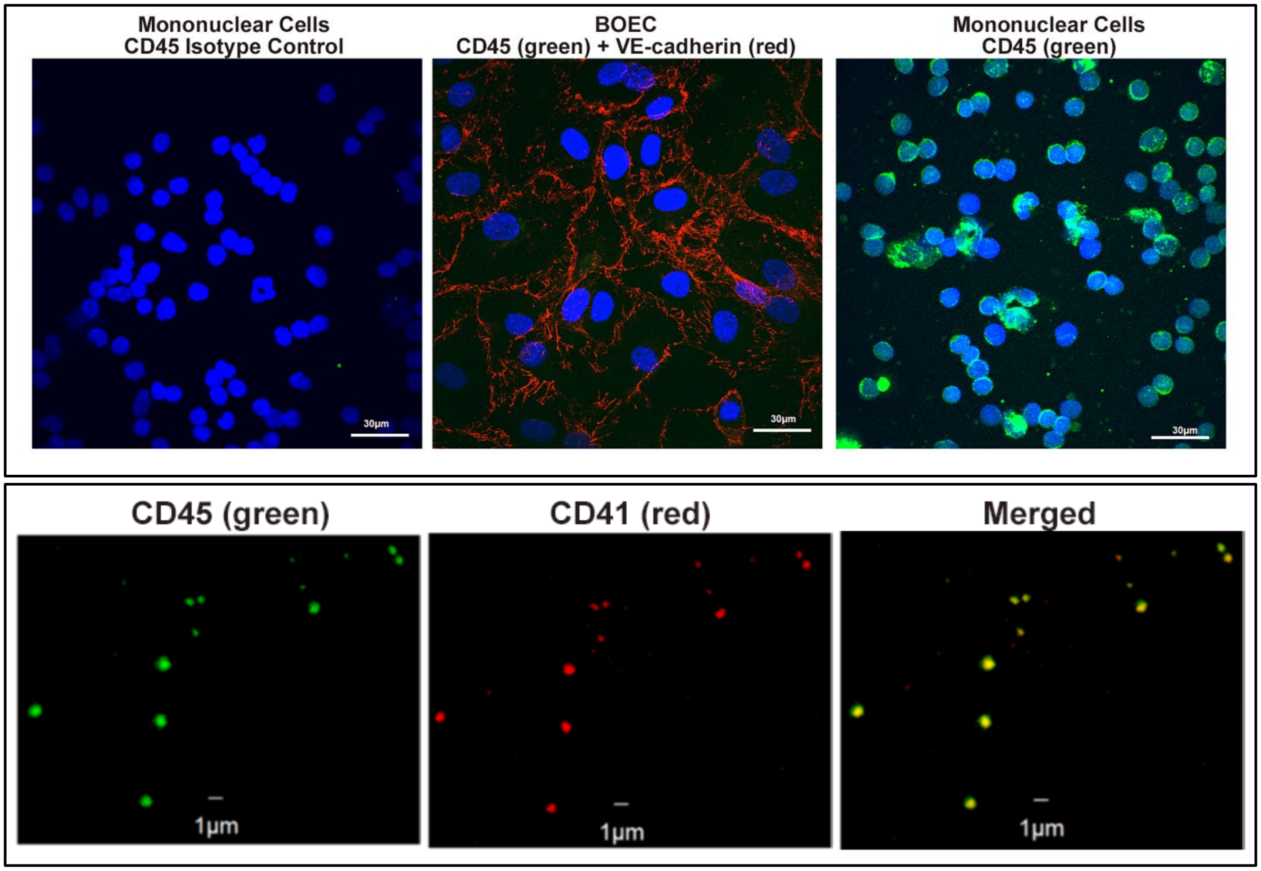

Supplemental Figure 7: CD45 antibody validation in mononuclear cells, BOEC, and platelet EVs. Upper panel. Human mononuclear cells or BOEC were stained with primary monoclonal antibodies (mAb) to CD45 (hematopoietic cell marker) and VE-cadherin (endothelial cell marker), followed by staining with a FITC (green)-labeled secondary antibody to the CD45 mAb and a PE (red)-labeled secondary antibody to the VE-cadherin mAb. Nuclei in all panels were stained blue with DAPI. Images were collected using fluorescence confocal microscopy at 60X. Lower panel. Platelet EVs were purified from human plasma using a Capturem™ spin column and dried onto a glass slide and immunofluorescently stained for CD45 and CD41 (platelet-specific marker) followed by the appropriate FITC (green)-labeled (CD45) or PE (red)-labeled (CD41) secondary antibodies. Images were visualized using fluorescence confocal microscopy at 120X. CD45 and CD41 stains were overlayed in the merged image.

Supplemental Figure 8

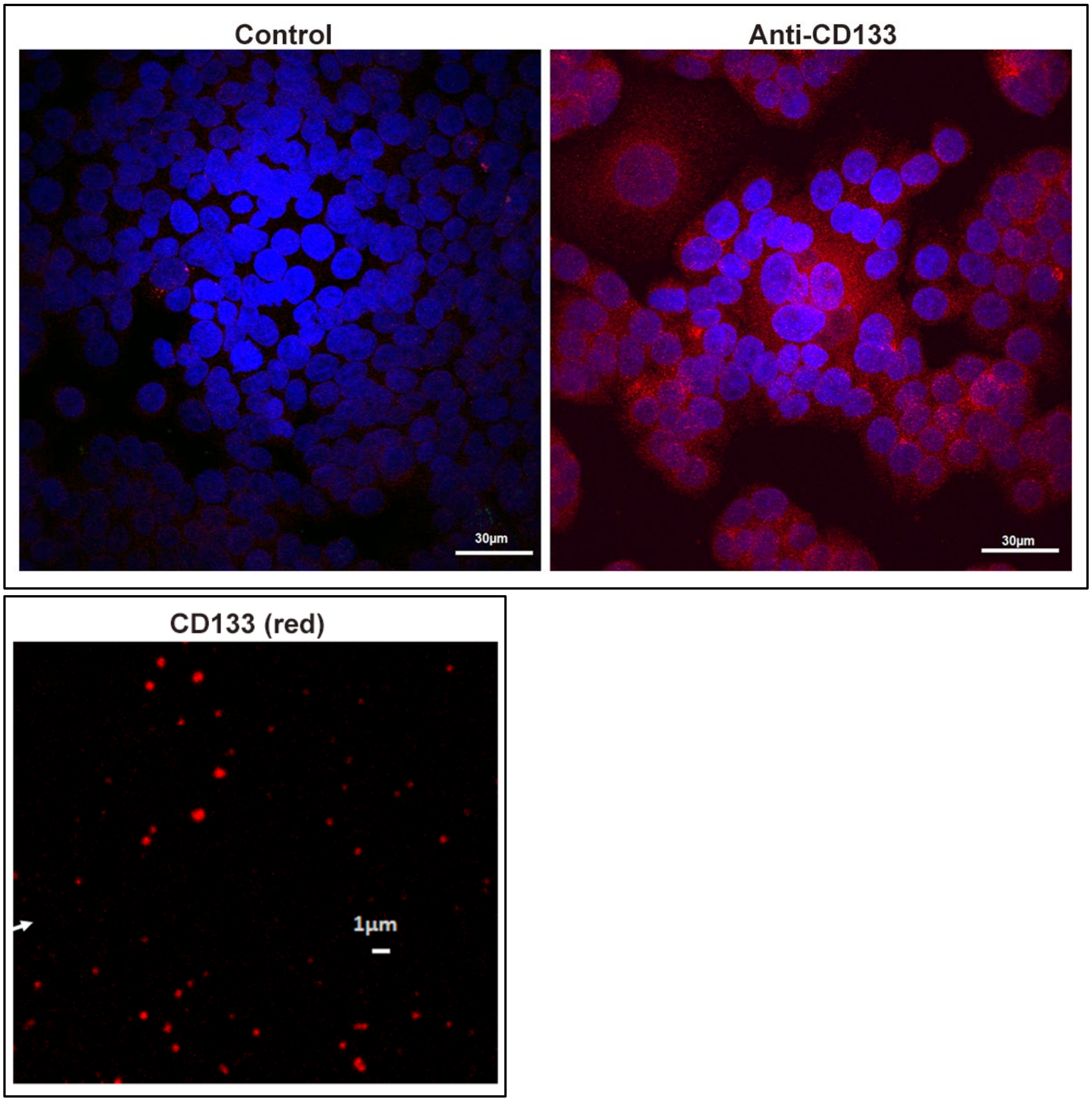

Supplemental Figure 8: Antibody Validation for CD133 on cells and EVs. Upper panel: CD133 (Prominin) is a marker for endothelial progenitor cells (EPCs). HT-29 cells in culture were immunofluorescently stained for CD133 followed by PE (red)-labeled secondary antibody. The control was HT-29 cells stained with PE-labeled secondary antibody without a primary MAb to CD133. Nuclei were stained blue with DAPI. Images were collected using fluorescence confocal microscopy at 60X. Lower panel: HT-29-conditioned media was collected after 72 hr and EVs were purified from the media using a Capturem™ spin column. Purified EVs were dried onto a glass slide and stained with a primary MAb to CD133 followed by the appropriate PE (red)-labeled secondary antibody to the primary MAb. Images were collected using fluorescence confocal microscopy at 120X.

Supplemental Figure 9

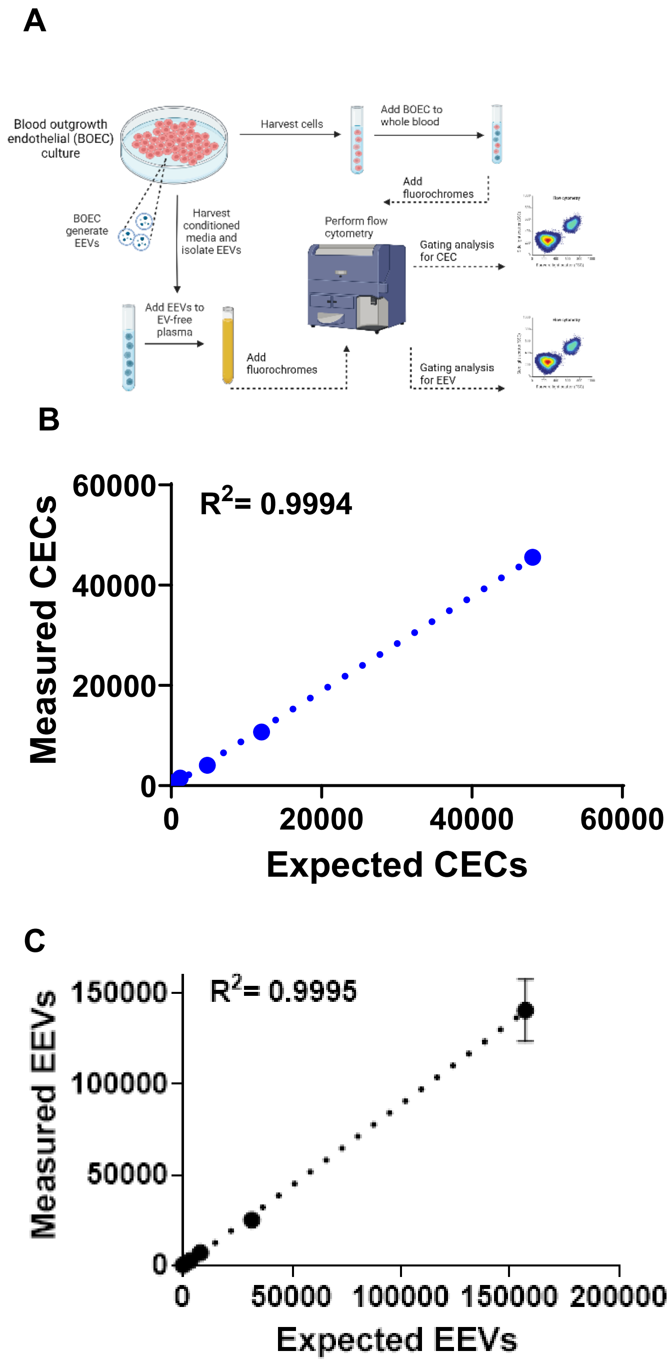

Supplemental Figure 9: Dynamic Range of CEC and EEV detection in whole blood and plasma. (A) Experimental protocol to determine dynamic range of the CEC and EEV assays using blood outgrowth endothelial cells (BOEC) and their EEVs added to control blood. Frozen BOEC (n=4/group) from a repository were thawed, cultured, and then trypsinized, counted, and added to blood samples from a control donor for CEC dynamic range testing. (B) Plot of measured versus anticipated CEC. The dotted line reflects simple linear regression. (C) Conditioned media from BOEC cultures were collected 72 hr after reaching confluency. EEVs from BOEC were isolated by ultracentrifugation at 120,000xg and enumerated by NTA. A known number of EEVs (n=4/group) were added to EEV-free platelet-free plasma (PFP) that was prepared by removing EEVs by ultracentrifugation at 120,000xg. Plot of measured versus anticipated EEV levels in PFP. The dotted line reflects simple linear regression.

Supplemental Figure 10

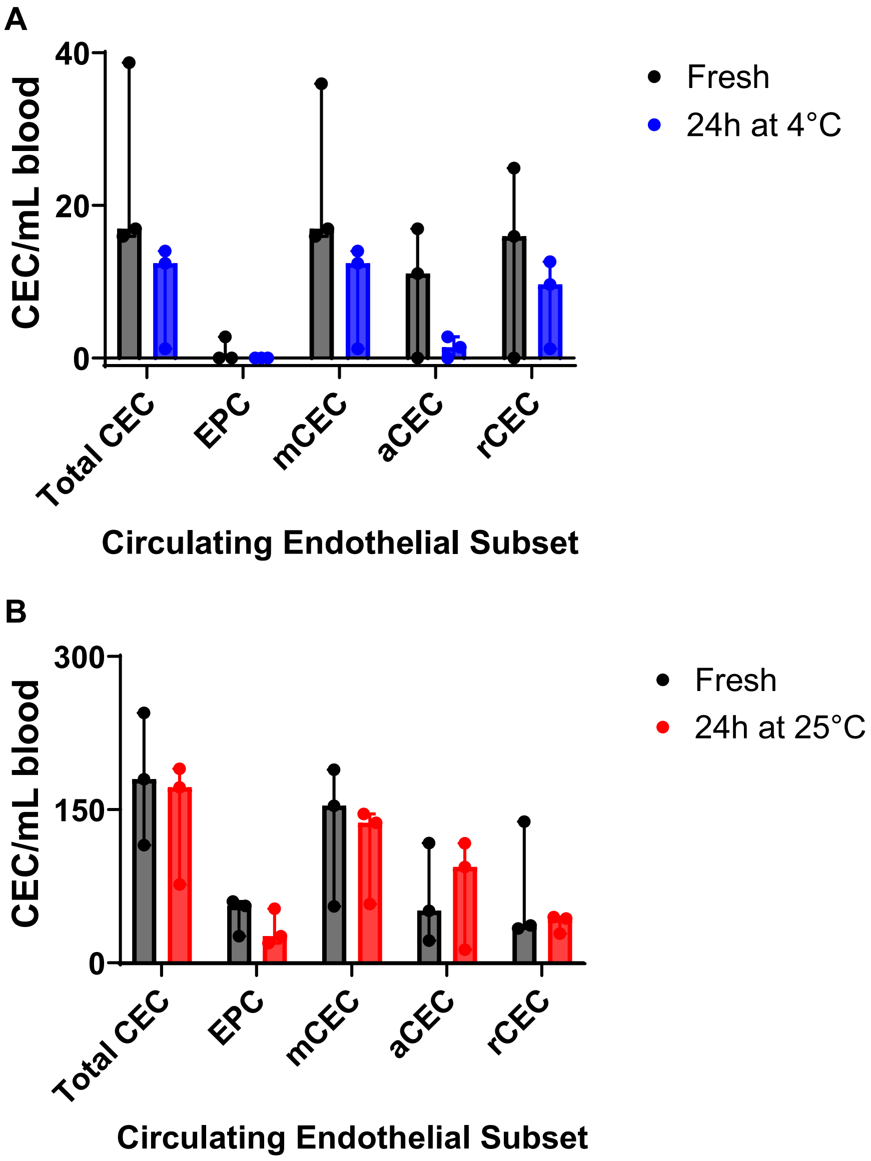

Supplemental Figure 10: Effects of blood storage on CEC populations: Circulating endothelial cell (CEC) populations were isolated from whole blood collected in EDTA. (A) Each blood sample (n=3) was divided into two aliquots. One aliquot was processed for CEC enumeration immediately (fresh) and the second was held at 4^0C for 24 h prior to CEC enumeration. (B) A separate cohort of blood samples (n=3) were collected in EDTA and divided into two aliquots. One aliquot was processed for CEC enumeration immediately (fresh) and the second aliquot was held at room temperature (25^0C) for 24 h prior to CEC enumeration. Values are median value +/- 95% confidence interval.

Supplemental Figure 11

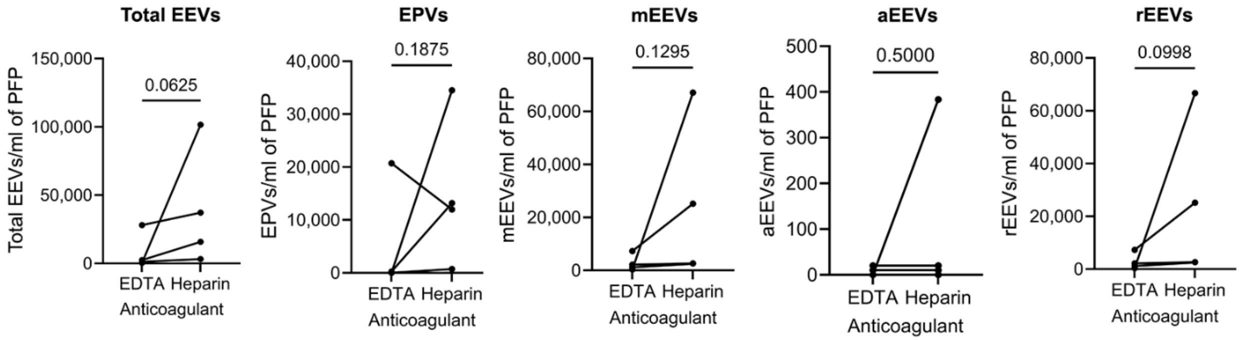

Supplemental Figure 11: Heparin anticoagulation appears to increase endothelial EEV numbers in PFP. Blood samples (n=4) were collected into EDTA and heparin anticoagulated vacutainer tubes from the same four individuals and processed into PFP. Next, EEV populations in PFP were stained and enumerated by flow cytometry. EDTA and Heparin means for each EEV subgroup were compared using the Wilcoxon matched-pairs signed rank test.

Supplemental References

1. Martin-Ramirez J, Hofman M, van den Biggelaar M, Hebbel RP, Voorberg J. Establishment of outgrowth endothelial cells from peripheral blood. *Nat Protoc*. 2012;7(9):1709-1715.
2. Wei P, Milbauer LC, Enenstein J, Nguyen J, Pan W, Hebbel RP. Differential endothelial cell gene expression by African Americans versus Caucasian Americans: a possible contribution to health disparity in vascular disease and cancer. *BMC Med*. 2011;9:2.
3. Beckman JD, Chen C, Nguyen J, et al. Regulation of heme oxygenase-1 protein expression by miR-377 in combination with miR-217. *J Biol Chem*. 2011;286(5):3194-3202.
4. Gaiser T, Camps J, Meinhardt S, et al. Genome and transcriptome profiles of CD133-positive colorectal cancer cells. *Am J Pathol*. 2011;178(4):1478-1488.
5. Zhou F, Zhou Y, Yang M, Wen J, Dong J, Tan W. Optimized multiparametric flow cytometric analysis of circulating endothelial cells and their subpopulations in peripheral blood of patients with solid tumors: a technical analysis. *Cancer Manag Res*. 2018;10:447-464.
6. Jayachandran M, Miller VM, Heit JA, Owen WG. Methodology for isolation, identification and characterization of microvesicles in peripheral blood. *J Immunol Methods*. 2012;375(1-2):207-214.
7. Wisgrill L, Lamm C, Hartmann J, et al. Peripheral blood microvesicles secretion is influenced by storage time, temperature, and anticoagulants. *Cytometry A*. 2016;89(7):663-672.
